# EGFR/FOXO3a/BIM signaling pathway determines chemosensitivity of BMP4-differentiated glioma stem cells to temozolomide

**DOI:** 10.1038/s12276-020-0479-9

**Published:** 2020-08-12

**Authors:** Iwona Anna Ciechomska, Bartlomiej Gielniewski, Bartosz Wojtas, Bozena Kaminska, Jakub Mieczkowski

**Affiliations:** Laboratory of Molecular Neurobiology, Neurobiology Center, Nencki Institute of Experimental Biology PAS, 3 Pasteur St, 02-093 Warsaw, Poland

**Keywords:** CNS cancer, Cancer stem cells, Cancer therapeutic resistance, Chemotherapy

## Abstract

Accumulating evidence suggests that glioma stem cells (GSCs), which are rare cells characterized by pluripotency and self-renewal ability, are responsible for glioblastoma (GBM) propagation, recurrence and resistance to therapies. Bone morphogenic proteins (BMPs) induce GSC differentiation, which leads to elimination of GSCs and sensitization of glioma to chemotherapeutics. Alterations in the epidermal growth factor receptor (*EGFR*) gene are detected in more than half of GBMs; however, the role of EGFR in the chemoresistance of GSCs remains unknown. Here, we examined whether EGFR signaling affects BMP4-induced differentiation of GSCs and their response to the alkylating drug temozolomide (TMZ). We show that BMP4 triggers the SMAD signaling cascade in GSCs independent of the EGFR level. BMP4 downregulated the levels of pluripotency markers (SOX2 and OLIG2) with a concomitant induction of an astrocytic marker (GFAP) and a neuronal marker (β-Tubulin III). However, GSCs with different EGFR levels responded differently to treatments. BMP4-induced differentiation did not enhance sensitivity to TMZ in EGFR^low^ GSCs, in contrast to EGFR^high^ GSCs, which underwent apoptosis. We then identified differences in cell cycle regulation. In EGFR^low^ cells, BMP4-triggered G1 cell cycle arrest which was not detected in EGFR^high^ cells. RNA-seq profiles further highlighted transcriptomic alterations and distinct processes characterizing EGFR-dependent responses in the course of BMP4-induced differentiation. We found that the control of BIM (the pro-apoptotic BCL-2 family protein) by the AKT/FOXO3a axis only operated in BMP4-differentiated EGFR^high^ cells upon TMZ treatment.

## Brain cancer: Stem cell properties may determine chemotherapy success

The properties of individual glioma stem cells (GSCs) may influence the success of chemotherapy in tackling aggressive brain cancer. GSCs promote tumor growth and chemotherapy resistance in glioblastoma tumors. One potential treatment approach uses bone morphogenetic proteins to induce GSCs to differentiate into less harmful cells. Once the GSC population has dwindled, chemoresistance reduces in many but not all cases. Jakub Mieczkowski, Bozena Kaminska and co-workers at the Nencki Institute of Experimental Biology in Warsaw, Poland, conducted experiments on patient-derived glioblastoma cell cultures. They found that samples with high expression levels of the epidermal growth factor receptor (EGFR) protein in GSCs showed heightened sensitivity to the chemotherapy drug temozolomide after differentiation. Conversely, low levels of EGFR resulted in chemoresistance being maintained after differentiation, which may explain the failure of chemotherapy in some patients.

## Introduction

Glioblastoma (GBM; World Health Organization grade IV glioma) originates from neural stem or progenitor cells, when they undergo oncogenic transformation. GBMs show a high recurrence rate, and their resistance to therapy results in short patient survival, despite aggressive treatment including surgical resection, radiotherapy, and chemotherapy with temozolomide (TMZ). Antitumor efficacy of TMZ is limited by O6-methylguanine-DNA-methyltransferase (MGMT), a DNA repair protein. Methylation of the *MGMT* promoter reduces *MGMT* expression and its DNA repair activity and increases sensitivity to TMZ^1,2^. Out of dozens of genetic defects resulting in disrupted signaling pathways in GBMs, alterations in the epidermal growth factor receptor (*EGFR*) gene are frequent in GBMs^3,4^. The downstream target of EGFR signaling, the PI3K/AKT axis, controls cell survival and cell death by negatively regulating apoptosis and the expression of *BCL2L11*, encoding BIM (the pro-apoptotic protein from the BCL-2 family)^5–7^. AKT phosphorylates Forkhead box O (FOXO) transcription factors, including FOXO3a, which is subsequently exported from the nucleus to inhibit FOXO3a-dependent transcription. Inactivation of AKT leads to FOXO3a dephosphorylation, nuclear translocation, and transcription of target genes that regulate proliferation, differentiation, and apoptosis^8^. Thus, aberrations in EGFR signaling may limit programmed cell death initiated by therapeutics.

GBMs are highly heterogeneous tumors containing cells with distinct functional phenotypes and different molecular abnormalities^9^. Primary GBMs are either mono- or polygenomic tumors (64 versus 36%, respectively) and express glioma stem cell markers, including CD133, CD15, A2B5, and CD44^10^. The presence of glioma stem cells (GSCs) or tumor initiating cells confers intrinsic GBM heterogeneity^11^. GSCs have a great impact on glioma progression and treatment response, as the failure of current therapies to eliminate GSCs is considered to be a major factor contributing to inevitable GBM recurrence. Therapeutics specifically targeting GSCs are proposed as a promising treatment strategy. One such approach is to differentiate GSCs to reduce their intrinsic resistance^12–14^.

Several studies have demonstrated the differentiating potential of bone morphogenetic proteins (BMPs) in GBM-derived stem cells. BMP2- or BMP7-mediated differentiation makes tumor cells more sensitive to chemotherapeutics^15,16^. BMP4 was shown to induce GSC differentiation^14,17^, although detailed molecular mechanisms of BMP4-induced differentiation and improved response to chemotherapy need to be elucidated. BMP proteins bind as homodimers to a receptor complex composed of two BMP type 1 receptors (BMPR1A or BMPR1B class) and two type 2 receptors (BMPR2). Ligand binding and receptor kinase activation leads to phosphorylation of SMAD 1/5/8 proteins, which translocate into the nucleus in a complex with SMAD4 and activate transcription of genes involved in stem cell differentiation^18–20^. Exit from the cell cycle and arrest at the G0/G1 phase are prerequisites for cell differentiation^21^. Proteins from the BMP family trigger G1 arrest via induction of cyclin-dependent kinase inhibitor 1A (*CDKN1A*, p21^CIP1^) and cyclin-dependent kinase inhibitor 1B (*CDKN1B*, p27^KIP1^)^22,23^.

We hypothesized that the differentiation of GSCs with BMP4 would enhance their sensitivity to TMZ. We used four patient-derived GBMs to generate GSC-enriched, sphere cultures with different EGFR levels, and then we characterized the steps of BMP4-activated differentiation. We found significant differences in the outcomes of the treatment depending on EGFR expression, particularly in the cell cycle distribution and expression of cell cycle-related proteins. Using RNA-seq, we found transcriptomic differences and distinct processes that distinguish the responses of EGFR^low^ and EGFR^high^ cells to BMP4-induced differentiation. The AKT/FOXO3a axis was an inducer of the pro-apoptotic protein BIM after TMZ treatment only in BMP4-differentiated EGFR^high^ cells. The results pinpoint the importance of tumor genetic diagnostics to improve the response to therapies.

## Materials and methods

### Reagents and antibodies

Reagent and antibody sources were as follows: AG1478 (Calbiochem/Merck, Darmstadt, Germany), BMP4 (R&D Systems, Minneapolis, MN, USA), DAPI (4′,6-diamidino-2-phenylindole dihydrochloride), MTT (3-(4,5-dimethylthiazol-2-yl)-2,5-diphenyltetrazolium bromide), temozolomide (TMZ) and anti-β-Actin-peroxidase conjugated antibody (Sigma-Aldrich, Munich, Germany), anti-AKT, anti-phospho-AKT (Ser473), anti-phospho-AKT (Thr308), anti-BIM, anti-cleaved caspase 3, anti-cleaved caspase 7, anti-cleaved PARP (poly (ADP-ribose) polymerase-1), anti-EGF Receptor, anti-phospho-EGF receptor (Tyr1068), anti-FOXO3a, anti-phospho-FOXO3a (Thr32), anti-phospho-FOXO3a (Ser253), anti-phospho-FOXO3a (Ser318/321), anti-phospho-Rb (Ser807/811), anti-SMAD1, anti-SMAD3, anti-SMAD4, anti-SMAD5, anti-phospho-SMAD1/5 (Ser463/465), anti-phospho-SMAD3 (Ser423/425), anti-p27^Kip1^, anti-SOX2 (Cell Signaling Technology, Beverly MA, USA), anti-OLIG2, anti-β-Tubulin beta III isoform (Millipore, Temecula, CA, USA), anti-CYCLIN B1, p21^CIP1^ (Santa Cruz Biotechnology, Dallas, Texas, USA), anti-GFAP (BD Pharmingen San Jose, CA), anti-NESTIN (R&D Systems, Minneapolis, MN, USA), and anti-CYCLIN D1 (ThermoFisher Scientific, Waltham, MA USA).

### Cell culture and differentiation induction

The L0125, L0512, L0615, and L0627 GBM GSC lines were provided by Dr Rossella Galli (San Raffaele Scientific Institute, Milan, Italy)^24–26^. For sphere forming assays, cells were seeded at a low density (3000 viable cells/cm^2^) onto nonadherent plates and cultured in DMEM/F-12 medium supplemented with 2% B27 (Gibco Invitrogen, Basel, Switzerland), 20 ng/ml rh bFGF (Miltenyi Biotec, Bergisch Gladbach, Germany), 20 ng/ml rh EGF (StemCell Technologies, Vancouver, BC, Canada), 0.0002% heparin (StemCell Technologies, Vancouver, BC, Canada), and antibiotics (Gibco Invitrogen, Basel, Switzerland). Cells were fed every 3 days by replacing 25% of the medium volume. After 6, 10, or 13 days of culturing, the spheres were collected by centrifugation at 110×*g* and then were lysed in Qiagen RLT lysis buffer for RNA isolation, lysed in buffer supplemented with complete protease inhibitor cocktail (Roche Applied Science, Indianapolis, IN, USA) for blotting, or the DNA was extracted.

For differentiation experiments, 6-day spheres were treated with 100 ng/ml BMP4 for 4 days with fresh BMP4 every 2 days. In some experiments, spheres were triturated to produce a single-cell suspension and then were seeded (2.5 × 10^4^ viable cells/cm^2^) onto laminin-coated plates in the medium as described above. The next day after seeding, the cells were exposed to 100 ng/ml BMP4 for 4 days (fresh BMP4 was added every 2 days). After treatment, cells were fixed with 4% paraformaldehyde (PFA) for immunocytochemistry, or they were collected for RNA and protein extraction.

For cell viability evaluation, 2 days after seeding as described above, adherent cells were exposed to various concentrations of AG1478 (0.25–5 µM) for 3 days.

### Cells treatments

Control (maintained without treatment for 6 days) or BMP4-differentiated spheres/cells were treated with 500 µM TMZ or 1 µM AG1478 for 3 days. As DMSO induced the differentiation of embryonic stem cells^27^, for most experiments, TMZ was dissolved in deionized water. In some experiments, TMZ was dissolved in DMSO, and DMSO was used at corresponding concentrations. Sphere formation and the effect of drugs on cell morphology were monitored using an Olympus X70.

### Immunoblotting

Whole cell lysates were prepared in a buffer containing phosphatase and protease inhibitors, and then they were separated by SDS-PAGE and transferred onto nitrocellulose membranes as described^28,29^. After blocking with 5% nonfat milk in a blocking buffer, the membranes were incubated overnight with primary antibodies and then with the appropriate secondary antibodies for 1 h. Immunocomplexes were visualized using an enhanced chemiluminescence detection system (SuperSignal West Pico PLUS, ThermoFisher Scientific, Waltham, MA, USA). The molecular weight of proteins was estimated with prestained protein markers (Sigma-Aldrich, Saint Louis, MO).

### MTT metabolism test

Cell viability was evaluated using MTT metabolism tests, as previously described^29^. Briefly, upon cell treatment, MTT stock solution was added at a final concentration of 0.5 mg/ml. After 1 h of incubation at 37 °C, water-insoluble formazan was dissolved in DMSO. Optical densities were measured at 570 nm using a scanning multiwell spectrophotometer.

### Flow cytometry

For cell cycle analysis, at least 0.5 × 10^6^ cells were used. GBM cells were triturated to produce a single cell suspension, and then they were resuspended in ice-cold PBS and fixed in 70% ethanol overnight at −20 °C. After centrifugation, the cell pellet was washed with PBS and then incubated for 15 min in the dark in propidium iodide/RNase staining buffer (BD Pharmingen San Jose, CA). Cells (2000) were analyzed by flow cytometry.

### Bisulfite DNA conversion and methylation-specific polymerase chain reaction (MS-PCR)

DNA was extracted using standard phenol/chloroform methods. The purity and concentration of DNA were estimated after collecting absorbance readings at 260/280 nm. DNA (2 μg) was treated with bisulfite (EpiTect Bisulfite Kit, Qiagen, Hilden, Germany). The modified DNA was amplified using primers specific for methylated or unmethylated *MGMT* gene promoters, as listed in Table S1. Each PCR mixture contained 1 μl of DNA, 500 nM of primers, 1x reaction buffer containing 1.5 mM MgCl_2_, and 1 U HotStarTaq DNA Polymerase and 250 mM dNTPs (Promega, USA). PCR was performed with thermal conditions as follows: 95 °C for 10 min, 45 cycles of 95 °C for 30 s, 57 °C for 30 s and 72 °C for 30 s with a final extension of 72 °C for 10 minutes. PCR products were visualized using 1.5% agarose gel, yielding a band of 81 bp for a methylated product and 93 bp for an unmethylated product. Positive methylated and positive unmethylated controls (EpiTect PCR Control DNA Set Qiagen, Hilden, Germany) were included.

### qRT-PCR

Total RNA was extracted using an RNeasy Mini kit (Qiagen, Hilden, Germany) and purified using RNeasy columns. The integrity of RNA was determined using an Agilent 2100 Bioanalyzer. For qRT-PCR, total RNA from cells was used to synthesize cDNA by extension of oligo(dT)_15_ primers with SuperScript reverse transcriptase (Thermo Fisher Scientific, Waltham, MA, USA). Real-time PCR experiments were performed in duplicate using a cDNA equivalent of 37.5 ng RNA in a 10-µl reaction volume containing 2x SYBR Green Fast PCR Master Mix (Applied Biosystems, Darmstadt, Germany) and a set of primers. SPP1 primers were from Qiagen (QT01008798, Hilden, Germany); sequences of other primers are listed in Table S1. Data were analyzed by the relative quantification method using StepOne Software (Applied Biosystems, Darmstadt, Germany). The expression of each product was normalized to that of 18S rRNA.

### mRNA library preparation and sequencing

For transcriptome analysis, equivalent amounts of RNA were used. In total, 16 strand-specific polyA-enriched RNA libraries were prepared using a KAPA Stranded mRNA Sample Preparation Kit according to the manufacturer’s protocol (Kapa Biosystems, Wilmington MA, USA). Briefly, mRNA molecules were enriched from 50 to 250 ng of total RNA using poly-T oligo-attached magnetic beads (Kapa Biosystems). The obtained mRNA was subsequently fragmented, and first-strand cDNA was synthesized using reverse transcriptase and random hexamers. Subsequent cDNA synthesis was performed to generate double-stranded cDNA (dsDNA). Adenosines were added to the 3′ ends of dsDNA, and adapters were ligated (adapters from NEB, Ipswich, MA, USA). Following adapter ligation, uracil was digested by a USER enzyme from NEB (Ipswich, MA, USA) in a loop structure of the adapter. Adapters containing DNA fragments were amplified by PCR using NEB starters (Ipswich MA, USA). Library evaluation was performed with an Agilent 2100 Bioanalyzer using an Agilent DNA High Sensitivity chip (Agilent Technologies, Ltd.) The mean library size was 300 bp. Libraries were quantified using a Quantus fluorometer and QuantiFluor double-stranded DNA System (Promega). Libraries were run in a rapid run flow cell and were paired-end sequenced (2 × 76 bp) with a HiSeq 1500 system (Illumina, San Diego, CA, USA).

### RNA-seq data alignment, processing, and analysis

The sequenced paired-end reads were mapped to the hg38 genome using tophat2 aligner v2.1.1 and the default parameters^30^. The expression estimates for each gene were obtained using the Bioconductor package edgeR^31^. TPM (transcripts per kilobase million) values were calculated and used to perform all visualizations and all analyses in addition to assessing differential expression. Genes that had significant (Benjamini and Hochberg-corrected *P* < 0.05) changes in their expression levels were identified as differentially expressed. The analyses of signaling pathways and transcription factor motifs were performed with enrichKEGG and GSEA functions, respectively, from the clusterProfiler^32^ Bioconductor package. The significance thresholds for the pathway and motif analyses were set to 0.01 and 0.05, respectively (Benjamini and Hochberg). To elucidate information about signal processing, only signaling pathways representing “Environmental Information Processing” and “Cellular Processes” in the KEGG database were analyzed. All gene ontology analyses were performed with the GO.db Bioconductor package and 0.01 significance threshold on Bonferroni corrected P-value (Fisher’s exact test). All statistical analyses were performed with R programming environment (http://r-project.org). The gene expression data can be downloaded from the NIH GEO database (GSE140441).

### Statistical analysis of biochemical data

Data were analyzed by Student’s t-test or ANOVA with Duncan’s post hoc test using R programming environment.

## Results

### BMP4 treatment triggers the differentiation of GBM-derived sphere cultures

**Fig. 1:**
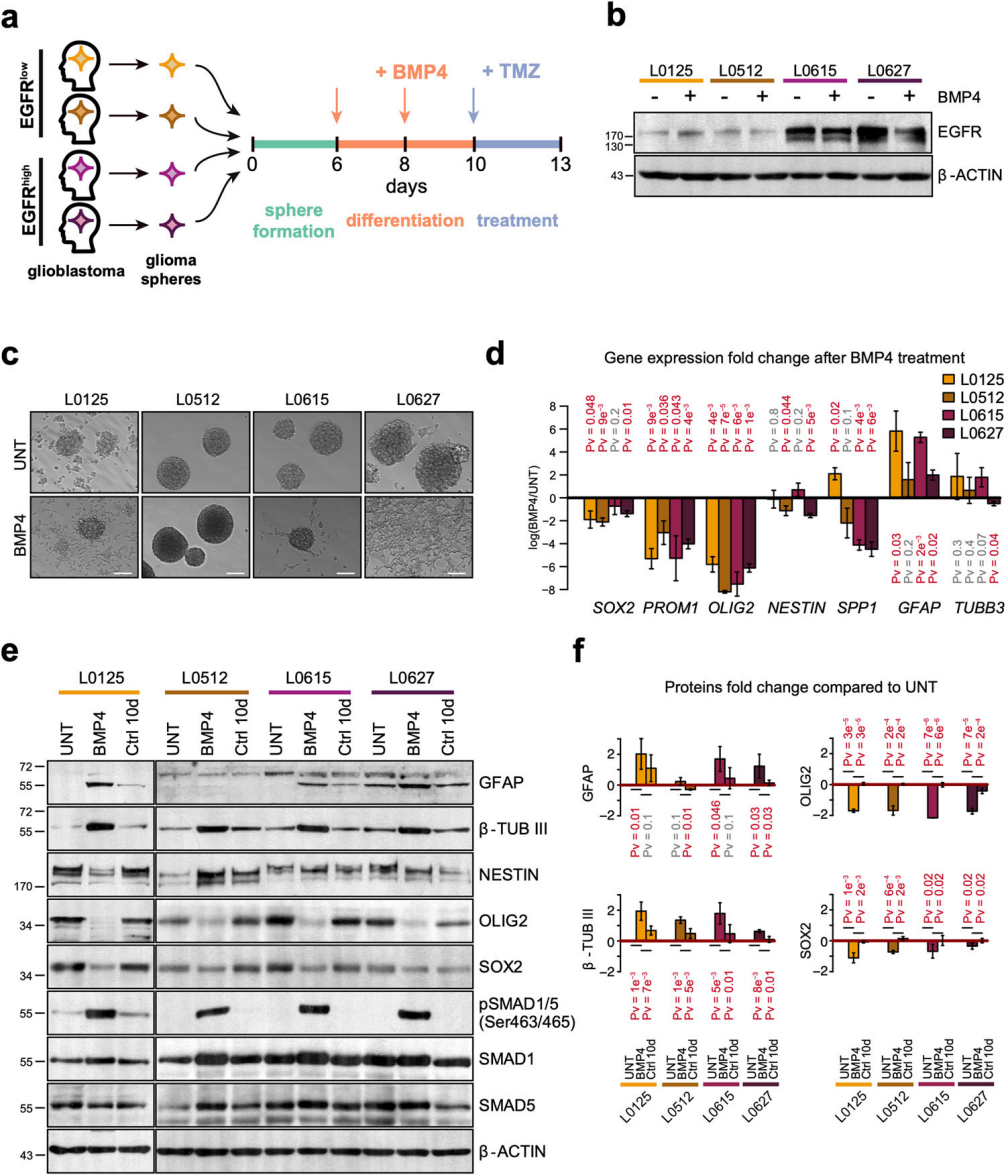
BMP4 treatment promotes the differentiation of glioma spheres. **a** Experimental scheme of treatments. Red and blue arrows indicate BMP4 and TMZ treatments, respectively. **b** Western blot analysis of EGFR in untreated and BMP4-treated glioma spheres. β-actin was used as a loading control. **c** Microphotographs of untreated (upper row) and BMP4-treated (bottom row) glioma spheres. Different cell lines are shown in columns and are indicated above the photographs. Scale bars represent 100 μm. **d** Quantitative PCR analysis of pluripotency markers (*SOX2, PROM1, OLIG2, NESTIN*, and *SPP1*) and differentiation markers (*GFAP* and *TUBB3*) in untreated and BMP4-treated glioma spheres. The results are presented as fold change in a log-scale. Gene expression in BMP4-treated cells was compared to untreated cells; data are presented as the mean ± SD of three independent experiments. Significance was tested with a *t* test, and computed *P*-values are indicated above the corresponding bars; *P* < 0.05 was considered significant. **e** Western blot analysis of selected pluripotency markers (NESTIN, OLIG2, and SOX2), differentiation markers (GFAP and β-TUBIII) and SMAD proteins. Untreated spheres maintained for 10 days (Ctrl 10 d) were used as controls. β-actin was used as a loading control. **f** Densitometric analysis of Western blots from three independent experiments (means ± SD, log-scale). The protein levels were normalized to β-actin levels and then to the corresponding levels in untreated spheres (UNT). The statistical significance was tested with ANOVA and Duncan’s post hoc test. The computed adjusted *P*-values are indicated above the corresponding bars. *P* < 0.05 was considered significant.

To determine whether BMP4-induced differentiation modulates GSC resistance to temozolomide, we performed experiments as depicted in Fig. 1a. The experiments were performed using four GBM patient-derived GSC cultures with different levels of EGFR; the cultures were maintained in serum-free medium as floating spheres. L0125 and L0512 cells had low EGFR expression (EGFR^low^), while L0615 and L0627 cells exhibited high EGFR levels (EGFR^high^). BMP4 treatment did not change the EGFR levels (Fig. 1b). The expression of BMP receptors was detected in all tested sphere cultures. The level of pathway-specific phospho-SMAD1/5, but not phospho-SMAD3, was increased upon BMP4 stimulation (Fig. S1). This indicates that BMP4 induces canonical BMP signaling in all tested GSCs and that different EGFR expression levels do not affect the SMAD signaling cascade (Fig. S1).

Upon BMP4 addition, most L0125 and L0627 cells showed signs of differentiation, and the cells became attached to the plates and flattened. L0615 spheres maintained their spherical shapes; however, the majority of spheres attached to the plates, and cells started to branch out (Fig. 1c). L0512 spheres were floating and maintained a spherical shape after BMP4 addition. The expression of pluripotency markers (*SOX2, PROM1, OLIG2*, and *SPP1*) significantly decreased in GSCs exposed to BMP4, with the exception of *SOX2* and *SPP1* (coding for osteopontin) in L0615 and L0125 sphere cultures, respectively (Fig. 1d). The expression of *NESTIN* (a marker of neural precursors) was significantly reduced only in L0512 and L0627 cells. Three out of four sphere cultures responded to BMP4 by increasing the mRNA levels of an astrocytic marker (glial fibrillary acidic protein*, GFAP*; Fig. 1d). The expression of a neuronal marker (β-Tubulin III, *TUBB3*) was not significantly increased by BMP4 treatment in L0125, L0512, and L0615 cells and was significantly downregulated in L0627 cells. However, western blot analysis indicated significant induction of β-Tubulin III in four cell lines, GFAP in three cell lines (except for L0512) and downregulation of OLIG2 and SOX2 in four cell lines (Fig. 1e, f). The expression of selected proteins remained unchanged in untreated sphere cultures maintained for 10 days, so the observed changes did not result from prolonged culturing (Ctrl 10 day, Fig. 1e, f). The most prominent effects were observed in BMP4-treated L0125 and L0615 GSCs; therefore, these two cell lines were used for global gene expression profiling.

### Treatment with BMP4 impairs TMZ-induced cytotoxicity in EGFR^low^ cells but not in EGFR^high^ cells

Using bisulfide methylation PCR, we found that L0125, L0512, and L0615 cells have both methylated and unmethylated *MGMT* gene promoters, whereas L0627 cells have only methylated promoters (Fig. S2a); therefore, L0627 cells should be more sensitive to TMZ. The cells were treated with TMZ for 72 h. We used TMZ dissolved in water, as the responses of GSCs to TMZ dissolved either in water or in DMSO were comparable (Fig. S2b–d). The analysis of the cell cycle and apoptotic hallmarks showed that independent of the solvent and *MGMT* status, TMZ-induced G2/M arrest and activation of the caspase cascade.

**Fig. 2:**
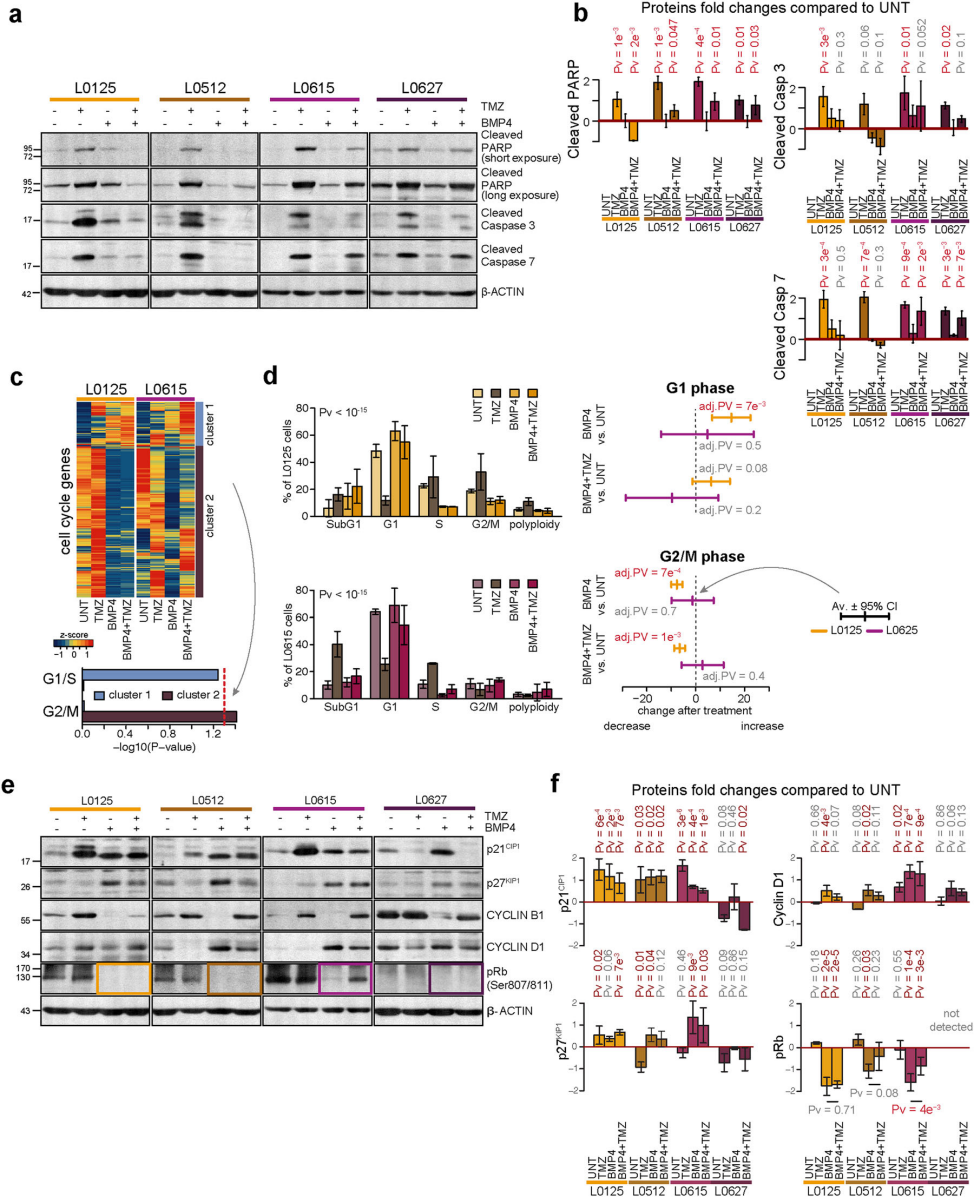
BMP4 treatment affects chemosensitivity in an EGFR-dependent manner. **a** Western blot analysis of biochemical markers of apoptosis. Immunoblots show the levels of cleaved caspase 3, caspase 7, and PARP (two exposures) in untreated, BMP4- and/or TMZ-treated glioma spheres. Detection of β-actin ensured equal protein loading. **b** Densitometric analysis of apoptotic markers (**a**). Quantifications of three (L0125 and L0615) or two (L0512 and L0627) independent experiments are shown. Data are presented as the means ± SD (log-scale). See the legend in Fig. 1f for more details. **c** Changes in the expression of cell cycle genes induced by BMP4 and/or TMZ treatments in L0125 and L0615 spheres. The heatmap (top) shows changes in the expression of the genes from the ‘cell cycle’ Gene Ontology category. *Z*-scores were computed separately for the two cell lines. An unsupervised clustering led to two main clusters, named cluster 1 and cluster 2, which are highlighted on the right side. The computed clusters showed associations with genes gathered as ‘regulation of cell cycle G1/S phase transition’ or ‘regulation of cell cycle G2/M phase transition’ Gene Ontology terms (bottom). *P*-values were computed using Fisher’s exact test. The red dotted line marks a *P*-value = 0.05. **d** Distribution of cells in cell cycle phases was determined by flow cytometry. The left panel presents the results for all cell cycle phases as the means ± 95% confidence intervals for the means computed from three independent experiments. *P*-values shown above the plots were computed using *χ*^2^ tests. The right panel shows the effects of selected treatments (BMP4 alone and BMP4 + TMZ) on the fraction of cells in G1 (top) or G2/M (bottom) phases. The effects are shown by the difference in means and 95% confidence intervals for the differences. Yellow and purple colors correspond to the L0125 and L0615 spheres, respectively. The statistical significance was tested with ANOVA and Duncan’s post hoc test. *P* < 0.05 is written in red. **e** Western blot analysis of cell cycle-related proteins in untreated and BMP4 ± TMZ-treated spheres. Levels of p21^CIP1^, p27^KIP1^, Cyclin B1, Cyclin D1, and phospho-Rb (Ser807/811) proteins were analyzed. β-actin was used as a loading control. **f** Densitometry analysis of immunoblots shown in **e**. Each bar and whisker represent the mean ± SD of two or three independent experiments (log-scale). The horizontal *P*-value corresponds to the comparison between BMP4- and BMP4 + TMZ-treated cells, which was computed with Duncan’s post hoc test.

We tested whether BMP4-induced differentiation sensitizes GSCs to TMZ. Surprisingly, pretreatment with BMP4 did not increase the expression of apoptotic markers and even reduced the TMZ effect on EGFR^low^ cells (Fig. 2a, b), as demonstrated by significantly lower levels of cleaved PARP and cleaved Caspases 3 and 7. Upon BMP4 and TMZ treatments, there was a reduction in apoptosis markers in EGFR^high^ cells compared to TMZ-treated cells, although possibly not to the same extent as what was observed in the EGFR^low^ cells (Fig. 2b). Because adherent GSCs are more frequently used for chemical screens than nonadherent GSCs^33^, we repeated the treatments using adherent GSC cultures. GSCs retained their stem properties for several days after adherence to the plates (data not shown). Adherent stem cells differentiated upon BMP4 exposure (Fig. S2e, f). However, even in adherent cultures, BMP4 treatment reduced sensitivity to TMZ in EGFR^low^ cells, in contrast to EGFR^high^ cells, which underwent apoptosis (Fig. S2e–g). These findings suggest that differences in the responses of BMP4-differentiated GSCs to TMZ may be intrinsic and may be associated with EGFR levels.

To explain the observed differences in the cells, we performed RNA-seq analysis of EGFR^low^ (L0125) and EGFR^high^ (L0615) spheres after BMP4- and/or TMZ- treatments. As both BMP4 and TMZ can influence the cell cycle, we compared the expression of genes involved in the cell cycle under the analyzed conditions (Fig. 2c). Using Gene Ontology (GO) annotations, we analyzed the expression changes of 916 genes grouped according to the ‘cell cycle’ GO term (GO:0007049). Their expression was different between EGFR^low^ and EGFR^high^ cells upon BMP4/TMZ treatment. Unsupervised clustering of the analyzed genes distinguished two main clusters. Treatment of BMP4-differentiated EGFR^low^ cells with TMZ did not change the expression of genes involved in the G2/M phase (cluster 2 on Fig. 2c, Pv = 0.977), while BMP4-differentiated EGFR^high^ cells showed increased expression of these genes after TMZ treatment (Fig. 2c, Pv = 0.038). This suggests that differences between EGFR^low^ (L0125) versus EGFR^high^ (L0615) cells in the cell cycle arrest were triggered by TMZ after BMP4-induced differentiation.

Flow cytometry analysis of the cell cycle phases revealed significant shifts between cell cycle phases in EGFR^low^ and EGFR^high^ cells after TMZ and/or BMP4 treatments (Fig. 2d, left panels). In EGFR^low^ cells, BMP4 treatment led to a significant increase in the number of cells in the G1 phase and a decrease in the number of cells in the G2/M phase (Fig. 2d, right panel). A decrease in the number of cells in G2/M phase was also observed after subsequent TMZ treatment (Fig. 2d, bottom right panel). In contrast, BMP4 treatment did not cause such changes in the cell cycle phase distribution in EGFR^high^ cells.

Furthermore, we analyzed the levels of cell cycle regulatory proteins, such as phospho-Rb (retinoblastoma), cyclin B1, cyclin D1, and two cyclin-dependent kinase inhibitors, p21^CIP1^ and p27^KIP1^ (Fig. 2e, f). Immunoblot analysis showed that p21^CIP1^ accumulated after BMP4, TMZ, and combined treatment. The highest level of p21^CIP1^ was detected in cells treated with TMZ alone, with the exception of L0627 cells, where a decreased level of p21^CIP1^ was observed. Accumulation of cyclin B1 was prominent in cells exposed to TMZ, which is consistent with G2/M arrest. Treatment with BMP4 significantly reduced the level of cyclin B1 (Fig. 2e). On the other hand, in three out of four GSC cultures, we found accumulation of cyclin D1 and p27^KIP1^ and reduction of phospho-Rb (Ser 807/811) after BMP4 treatment, indicating cell cycle arrest at the G0/G1 phase. In all cells in which phospho-Rb levels were detectable, BMP4 treatment significantly decreased the phospho-Rb level. However, the results of the combined treatment were different depending on EGFR signaling. Compared to TMZ treatment alone, the combined treatment did not affect phospho-Rb levels in EGFR^low^ cells, while in EGFR^high^ L0615 cells, a less prominent reduction in phospho-Rb was observed. In L0627 EGFR^high^ cells, the phospho-Rb levels did not increase under any condition, which suggests more complex regulation in those cells (Fig. 2e, f). These findings are in accord with the results of transcriptomic and flow cytometry analyses. We conclude that BMP4 treatment of GSCs triggers cell cycle arrest, but the outcome may be influenced by EGFR status.

### EGFR signaling affects BMP4-mediated differentiation

**Fig. 3:**
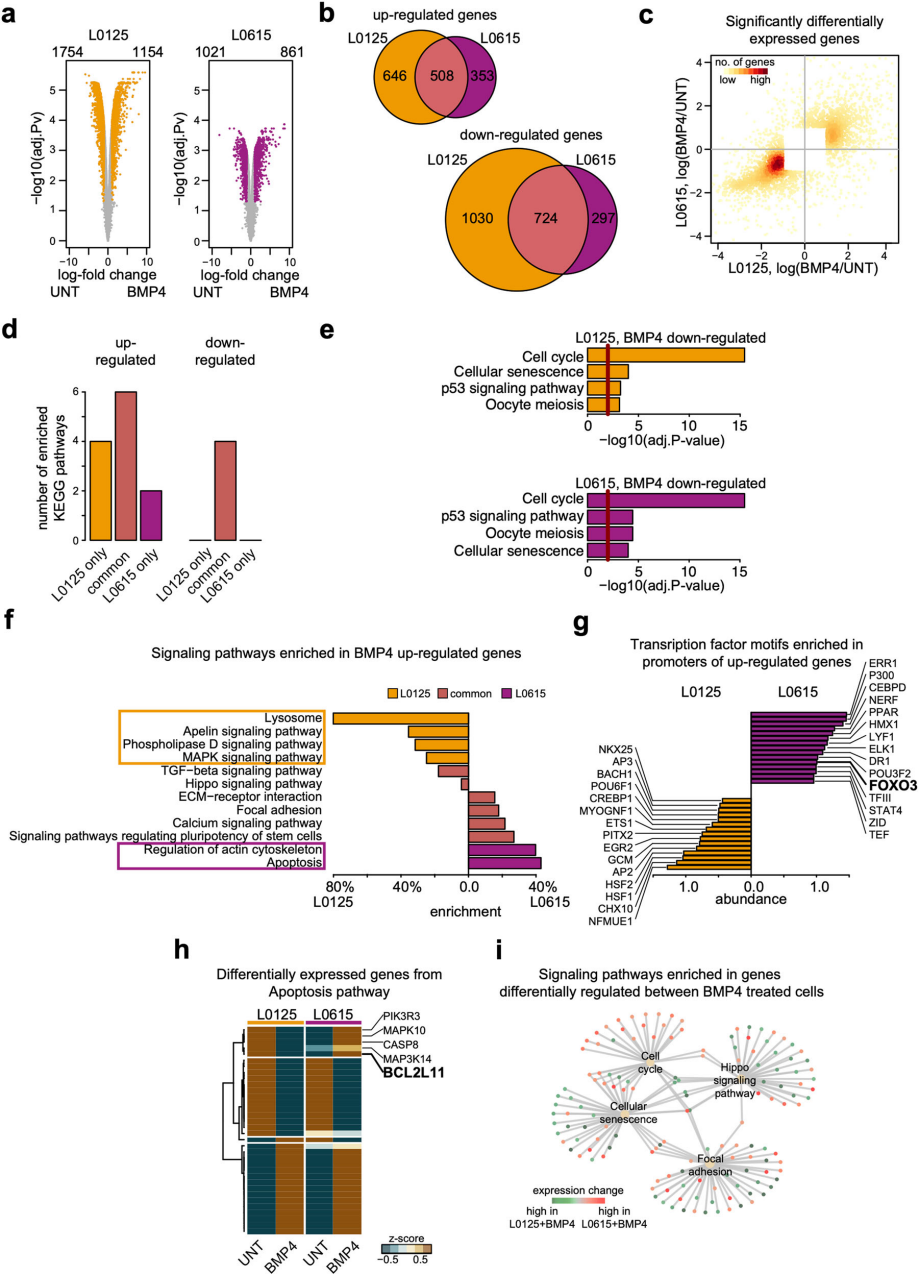
Global transcriptomic analysis reveals that apoptotic signaling differs in EGFR^low^ and EGFR^high^ cells after BMP4 treatment. **a** Comparison of gene expression profiles between untreated (two replicates) and BMP4-treated (two replicates) cells. The left and right panels correspond to L0125 and L0615 glioma spheres. Each point on the plots corresponds to one gene. The *X*-axis shows the expression ratio (BMP4/UNT, log-scale). The *Y*-axis presents the corresponding adjusted *P*-values computed with DESeq, -log10 (adj. *P*-value). The Benjamini and Hochberg method was applied for the *P*-value adjustment. Genes whose expression levels were significantly altered (adj. *P*-value<0.05 and |log fold change | >2) are marked with colors – yellow for L0125 cells and purple for L0615 cells. The numbers of genes significantly down- or upregulated are written above the plots. **b** Venn diagrams summarizing the overlap between differentially up- (top) and downregulated (bottom) genes in the two different cell lines. Yellow and purple circles represent L0125 and L0625 cell lines, respectively. **c** Scatter plot comparing gene expression changes of significantly differentially expressed genes after BMP4 treatment. Each point on the plot corresponds to one gene. Only genes differentially expressed in at least one cell line are plotted. The *X*-axis and *Y*-axis correspond to expression changes in the L0125 and L0615 cell lines, respectively. The color magnitude represents the density of points. **d** Number of KEGG signaling pathways significantly enriched in the sets of differentially downregulated or upregulated genes. The bars on the left correspond to sets of upregulated genes, while the bars on the right correspond to sets of downregulated genes. Yellow and purple bars depict results specific for the L0125 and L0615 cell lines, respectively. Light purple bars (second and fifth from the left) show a number of nonspecifically enriched pathways. **e** All KEGG pathways enriched in sets of genes significantly downregulated after BMP4 treatment. The top and bottom panels correspond to the L0125 and L0615 cell lines, respectively. *X*-axes on both plots represent Benjamini and Hochberg adjusted *P*-values. The red lines mark *P*-values = 0.05. **f** Significantly enriched signaling pathways in sets of upregulated genes upon BMP4 treatment. Yellow and purple bars depict results specific for the L0125 and L0615 cell lines, respectively. Names of pathways specifically enriched for either L0125 or L0615 cells are marked with rectangles. The bars represent relative enrichment (see methods for details). Signaling pathway definitions were obtained from the KEGG database. **g** Top transcription factors for which binding motifs in promoters of BMP4 upregulated genes were identified. Yellow and purple bars indicate the results for the L0125 and L0615 cell lines, respectively. The lengths of the bars represent log-odds difference of computed scores for the analyzed motifs. The analysis was performed using the Molecular Signatures Database. **h** A heatmap represents average expression changes of genes involved in apoptosis and differentially expressed after BMP4 treatment in at least one cell line. The *z*-scores were computed separately for the two cell lines and used to compute the average values (two replicates per average). The first two columns (from the left) correspond to the L0125 line, and the last two correspond to the L0615 line. The rows were clustered using an unsupervised method. **i** Networks visualizing signaling pathways enriched in a set of genes differentially regulated between BMP4-treated L0125 and L0615 cell lines. The pale yellow points depict identified pathways. Green and red dots represent genes with significantly higher expression levels in L0125 + BMP4 and L0615 + BMP4 cells, respectively. The gray lines represent genes that are members of a particular pathway.

To characterize molecular events in BMP4-differentiated EGFR^low^ and EGFR^high^ GSCs, we performed global gene expression profiling. First, we identified differentially expressed genes (DEGs) between untreated and BMP4-treated cells. BMP4 treatment caused greater transcriptomic changes in EGFR^low^ cells than it did in EGFR^high^ cells (Fig. 3a, left panel). In both cell types, more genes were downregulated than upregulated. The majority of genes downregulated in EGFR^high^ cells were also downregulated in EGFR^low^ (~71%). For upregulated genes, the similarity was less pronounced; only 59% of genes upregulated in EGFR^high^ were also upregulated in EGFR^low^ spheres (Fig. 3b). We used a scatterplot to directly compare changes in the expression levels of the identified DEGs (Fig. 3c). The fold changes of the expression of downregulated genes were more similar between cell types than the fold changes of upregulated genes. These results suggest that BMP4 differentiation causes broader expression changes in EGFR^low^ spheres, and downregulated processes in EGFR^low^ and EGFR^high^ cells are more similar than the upregulated ones.

To recognize functional processes modified by BMP4 treatment, we performed analysis of signaling pathways defined by Kyoto Encyclopedia of Genes and Genomes (KEGG, see methods for details). Interestingly, BMP4-triggered differentiation led to downregulation of the same signaling pathways in L0125 and L0615 glioma spheres (Fig. 3d, e). The strongest downregulation was observed in a pathway describing the cell cycle, confirming the findings presented above (Fig. 3e). Gene Ontology (GO) analysis supported these results. The top GO terms enriched in downregulated genes included DNA replication and mitotic division (Fig. S3a, b). These results reflect the inhibition of cell proliferation and again highlight that BMP4 triggered the differentiation of GSCs. On the other hand, the KEGG pathways enriched in upregulated DEGs provided a clear distinction between EGFR^low^ and EGFR^high^ cells (Fig. 3f). In accordance with our biochemical results (Fig. 1e, f), BMP4 activates elements of the TGF-β pathway in both EGFR^low^ and EGFR^high^ spheres. Additionally, a signaling pathway regulating the pluripotency of stem cells, which forms a part of the TGF-β pathway, was activated in both types of spheres. However, we observed that in EGFR^high^ spheres but not in EGFG^low^ spheres, BMP4-induced differentiation was sufficient to activate genes in the apoptotic pathway (Figs. 3f and S3c).

This result was supported by Gene Set Enrichment Analysis (GSEA) of transcription factor (TF) binding motifs. We identified several TFs that may be associated with the diverse response of GSCs to BMP4. Among TFs potentially involved in BMP4-induced upregulation in EGFR^high^ spheres but not in EGFR^low^ spheres, we identified FOXO3 (Fig. 3g). This protein can be regulated by EGFR signaling and triggers BIM-mediated apoptosis^34^. Therefore, we looked in greater depth at the genes involved in apoptotic pathways and differentially expressed after BMP4 treatment in at least one of the cell types. A vast majority of the selected genes showed the same expression profile between EGFR^low^ and EGFR^high^ cells (Fig. 3h). However, 5 genes, namely, *PIK3R3, MAPK10, CASP8, MAP3K14*, and *BCL2L11* (encoding BIM protein), were upregulated in EGFR^high^ cells and downregulated in EGFR^low^ cells.

The differences between BMP4-triggered differentiation in EGFR^low^ and EGFR^high^ cells were confirmed when the corresponding expression profiles were compared (Fig. 3i). In EGFR^high^ differentiated cells, we observed upregulation of genes grouped into a cell cycle pathway (adj. Pv = 0.0017). We also observed differential activation of focal adhesion (adj. Pv = 0.0029) and Hippo signaling pathway genes (adj. Pv = 0.0029), which were upregulated when compared to untreated cells. Additionally, we found stronger activation of cellular senescence signaling in EGFR^low^ cells than in EGFR^high^ cells (Fig. 3i, adj. Pv = 0.0013).

### BMP4 and TMZ activate the AKT/FOXO3a/BIM axis in an EGFR-dependent manner

**Fig. 4:**
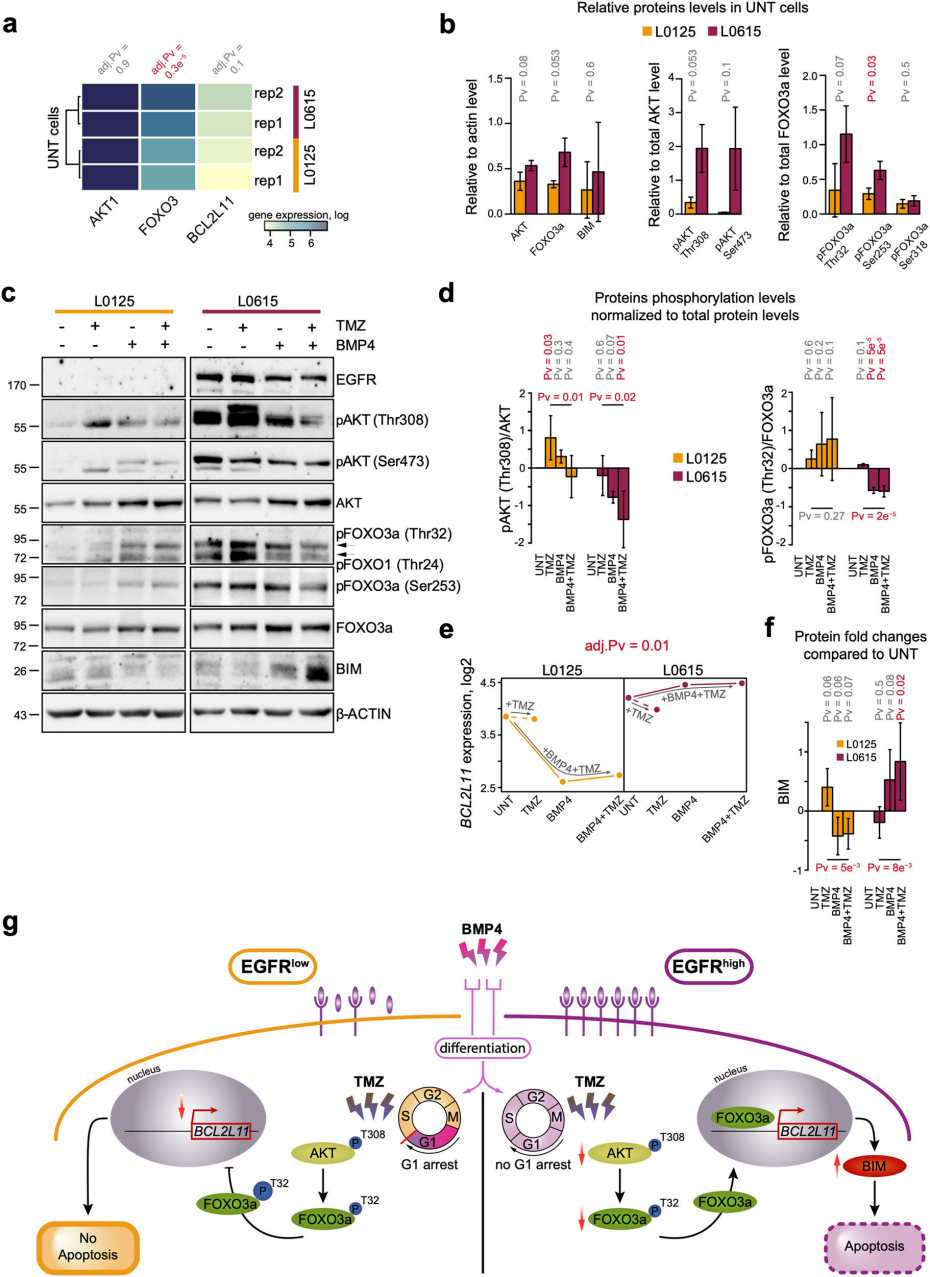
Involvement of the AKT/FOXO3a/BIM pathway in TMZ-induced apoptosis of BMP4-differentiated EGFR^high^ GSCs. **a** A heatmap representing RNA-seq expression of *AKT1*, *FOXO3*, and *BCL2L11* genes in EGFR^low^ (L0125, yellow) and EGFR^high^ (L0615, purple) untreated glioma spheres. The results are presented on log scale. *P*-values shown above the heatmap were computed using the DESeq method and adjusted with the Benjamini and Hochberg method. The result of unsupervised clustering is shown on the right. **b** A densitometric analysis of immunoblots (**c**) shows the total and phospho-AKT (Thr308 and Ser473), total and phospho-FOXO3a (Thr32, Ser253, and Ser318) and BIM levels in untreated L0125 and L0615 spheres. Left, middle, and right panels correspond to total, phospho-AKT, and phospho-FOXO3a protein levels, respectively. Each bar and whisker represent the mean ± SD of three independent experiments. The total protein levels were normalized to those of β-actin, while the levels of phosphorylated proteins were normalized to the total level of the corresponding protein. Statistical analysis is shown in Fig. 1. **c** Western blot analysis of EGFR, total- and phospho-AKT, total- and phospho-FOXO3a and BIM levels upon exposure to TMZ, BMP4, or both drugs. β-actin was used as a loading control. **d** Densitometric analysis of phospho-AKT (Thr308) and phospho-FOXO3a (Thr32). Each bar and whisker represent the mean ± SD of three independent experiments (log-scale). The levels of phosphorylated proteins were normalized to the corresponding total protein levels. See legend for Fig. 1f for more details. The horizontal *P*-values correspond to comparisons between TMZ- and BMP4 + TMZ-treated cells and were computed with the post hoc test. **e** *BCL2L11* gene expression in EGFR^low^ and EGFR^high^ cells after the treatments. The left and right panels correspond to L0125 and L0615 glioma spheres, respectively. The expression estimations are average values from the RNA-seq data of two independent experiments. The dashed lines correspond to TMZ treatment alone, while the solid lines correspond to pretreatment with BMP4 and subsequent treatment with TMZ. The adjusted *P*-value shown above the panels was computed using DESeq and was adjusted using the Benjamini and Hochberg method. **f** Densitometric analysis of BIM protein levels (*n* = 3). The bars and whiskers represent the means and SD (log-scale). The total protein levels were normalized to those of β-actin. See legend for Figs. 1f and 4d for more details. **g** A schematic summary of the obtained results. The left and right panels correspond to EGFR^low^ and EGFR^high^ glioma spheres, respectively. In EGFR^low^ cells, BMP4 stimulation triggers G1 cell cycle arrest, which leads to low *BCL2L11* expression and blocks TMZ-triggered cell death. In contrast, TMZ treatment of BMP4-differentiated EGFR^high^ cells upregulates the level of the pro-apoptotic protein BIM via AKT inactivation, increases FOXO3a dephosphorylation and promotes its translocation into the nucleus.

We compared the expression of *BCL2L11*, *FOXO3*, and *AKT1* in untreated cells (Fig. 4a) and found that neither *AKT1* nor *BCL2L11* showed differential expression. Only *FOXO3* was significantly upregulated in EGFR^high^ GSCs. Additionally, some differences in the levels of phosphorylated proteins were observed (Fig. 4b). However, BMP4 and TMZ treatments triggered distinct regulation of the AKT/FOXO3a/BIM proteins (Figs. 4c and S4a, b). BMP4 treatment did not significantly diminish the levels of AKT or FOXO3a phosphorylation in EGFR^low^ cells (Fig. 4d), and it did not increase phospho-FOXO3a (Ser318) levels (Fig. S4b). At the same time, in EGFR^high^ spheres, the levels of phosphorylated AKT (Thr308) and phosphorylated FOXO3a (Thr32) were significantly reduced (Fig. 4d). This was supported by enhanced levels of total AKT and FOXO3a after BMP4 treatment (Figs. 4d, S4a).

The changes in the mRNA and protein levels of *BCL2L11*/BIM corroborated our previous observations. The treatments led to significantly different regulation of the *BCL2L11* gene in the analyzed GSCs (Fig. 4e). The BIM level was significantly higher in BMP4-differentiated and TMZ-treated EGFR^high^ cells than it was in untreated cells or cells treated with TMZ alone (Fig. 4f). This suggests that inhibition of the AKT/FOXO3a axis leads to the preferential accumulation of BIM in EGFR^high^ cells (Fig. 4d, f). TMZ did not affect the expression of *BCL2L11* or the BIM protein level in BMP4-differentiated or undifferentiated EGFR^low^ cells (Fig. 4e, f). Similar changes in the AKT/FOXO3a/BIM pathway were observed in EGFR^low^ L0512 and EGFR^high^ L0627 cells (Fig. S4c, d). This result was in agreement with the analysis of the levels of biochemical markers of apoptosis, such cleaved Caspases and cleaved PARP (Fig. 2a, b).

To explore the contribution of EGFR signaling to the observed events, the effects of pharmacological inhibition of EGFR on the TMZ response of BMP4-differentiated cells were analyzed (Fig. S4e–g). AG1478 (EGFR tyrosine kinase inhibitor) efficiently blocked EGFR activation solely in EGFR^high^ cells (L0615 and L0627), as shown by a reduction in phospho-EGFR levels (Fig. S4e). Consistently, decreases in cell survival were observed only in EGFR^high^ cells exposed to AG1478 (Fig. S4f). Treatment with 1 µM AG1478 did not affect the downstream signaling pathways involving phospho-AKT and phospho-FOXO3a (Fig. S4g) and did not augment the expression of apoptotic markers in comparison to what was observed in BMP4 and TMZ treatments, as revealed by analysis of cleaved Caspases, cleaved PARP and BIM. The accumulation of BIM and cleaved PARP in L0615 cells upon treatment with AG1478 suggests that these cells are more sensitive to the EGFR inhibitor than the other cells tested (Fig. S4g).

Together, these findings indicate that functionality of the BIM-related apoptotic axis in BMP4-differentiated GSCs is triggered by TMZ but can be modified by signaling proteins downstream of EGFR (Fig. 4g).

## Discussion

Glioma stem cells form a rare cell population within a tumor, and their resistance to treatment contributes to tumor recurrence^35,36^. Patient-derived GSCs cultured as spheres reproduce the genetic and phenotypic characteristics of GBM more faithfully than established glioma cell lines^37^. In this study, for the first time, we explored whether EGFR signaling has an impact on the responses of differentiated GSCs to temozolomide, a drug of choice in glioblastoma therapy. We found that BMP4 treatment induces the differentiation of GSCs regardless of their EGFR status (Fig. 1), which is in line with recent reports^14^. BMP4 reduced the stemness features of GSC spheres and induced the expression of differentiation markers. Differences in the pattern of markers and degree of differentiation could be due to epigenetic characteristics of the individual tumor^38^ or secretion of BMP antagonists, which may determine responses of GSCs to differentiation-inducing agents^39,40^. Additionally, increased activity or levels of SOX transcription factors may limit differentiation commitment^13^.

Components of the BMP signaling pathway were expressed in four GSC sphere cultures, and their activation was independent of EGFR levels (Figs. 1 and S1). Different BMPs have been shown to deplete the GSC population without inducing cell death^14,41^. We found that BMP4 did not induce apoptosis in GSCs; it increased the number of EGFR^low^ GSCs in the G1 phase and decreased those in the G2/M phase (Fig. 2a–d). BMP4 treatment did not cause significant changes in the cell cycle phase distribution in EGFR^high^ GSCs (Fig. 2d). Carèn et al.^13^ reported that EGF/FGF signaling in GSCs differentiated with BMPs may trigger re-entry into the cell cycle. EGFR triggers downstream signaling pathways such as PI3K/AKT and Ras/Raf/MEK, which stimulate mitosis^42^. EGFR activation promotes glioma cell proliferation^43^, which may explain differences in the cell cycle arrest observed in cells with various EGFR levels. However, accumulation of two cyclin-dependent kinase inhibitors, p21^CIP1^ and p27^KIP1^, with concomitant reduction of phospho-Rb, was found in both cell types (Fig. 2e, f). The recognized differences in the expression of genes and proteins involved in cell cycle regulation were confirmed by genome-wide transcriptomic analysis (Fig. 2c). Interestingly, we found stronger activation of cellular senescence signaling in EGFR^low^ cells than we did in EGFR^high^ cells (Fig. 3i).

BMP2 and BMP7 have been shown to induce the differentiation of GBM-derived stem cells and to sensitize cells to TMZ^15,16^. Herein, we found that pretreatment with BMP4 did not enhance sensitivity to TMZ in EGFR^low^ GSCs, but BMP4-differentiated EGFR^high^ GSCs maintained the ability to undergo apoptosis (Fig. 2a, b). Sachdeva et al.^44^ reported that BMP pathway activation mediates GSC quiescence and confers resistance to radiation and TMZ chemotherapy. Although the status of EGFR was not analyzed, the effect of BMP4 on glioma cells was mediated by its downstream targets, p21^CIP1^ and ID1 (DNA-binding protein inhibitor 1).

The responses of different GSCs to TMZ could be variable for multiple reasons. It has been reported that GSCs derived from distinct tumor areas respond differently to TMZ. The cells from the inner portions of the GBM mass were more resistant to TMZ because of the high MGMT expression^15,45^. BMPs can sensitize GSCs to TMZ by affecting HIF1α stability and MGMT expression^15,16^. We demonstrated that neither TMZ nor BMP4 affected *MGMT* gene expression in L0125 cells, and in L0615 cells, the *MGMT* level was undetectable (data not shown). In our study, three out of four cell lines contained methylated and unmethylated *MGMT* gene promoters; the *MGMT* promoter in L0627 cells was found to be methylated (Fig. S2a), which was in accordance with other studies^46^. MGMT status had no impact on the responses of BMP4-differentiated EGFR^low^ and EGFR^high^ GSCs to TMZ.

Global transcriptomic analysis revealed that while BMP4 downregulated genes did not depend on EGFR expression, the genes and processes activated after BMP4 stimulation differed between EGFR^high^ and EGFR^low^ cells. Specifically, we found that AKT/FOXO3a/BIM signaling was differentially regulated in EGFR^high^ and EGFR^low^ GSCs after the combined treatment (Figs. 3, 4). The high level of AKT-dependent signaling in EGFR^high^ cells was independent of PTEN (phosphatase and tensin homolog deleted on chromosome 10) status, as described^26^. Several studies have reported that FOXO factors can initiate apoptosis by activating the transcription of *FasL*, coding for a ligand for the Fas-dependent cell death pathway, or *BIM*, a pro-apoptotic Bcl-2 family member^47^. BIM, as an activator BH3-only protein, binds to mitochondrial membranes and increases their affinity for pore-formers (e.g., BAX and BAK), which instigates mitochondria-dependent events such as the release of cytochrome c and other apoptotic factors^48^. Based on our findings, we assumed that TMZ induces BIM-dependent apoptosis in BMP4-differentiated EGFR^high^ GSCs. Intriguingly, we observed the proapoptotic effect of TMZ on undifferentiated GSCs; however, upon BMP4 differentiation, only EGFR^high^ cells responded in such a manner. One explanation could be that BMP4-induced G1 cell cycle arrest and resulting cellular senescence may lead to chemoresistance to TMZ in EGFR^low^ cells. In contrast, in EGFR^high^ GSCs, EGFR signaling caused re-entry of differentiated cells into the cell cycle. Actively proliferating cells are more prone to DNA damage-triggered cell death induced by alkylating agents such as TMZ. FOXO3a has been shown to cooperate with SMAD2/3 and mediate *BIM* upregulation and apoptosis in hepatocarcinoma cells upon TGF-β-stimulation^49^.

Our findings are of clinical importance and may explain failures of TMZ therapy in some GBM patients. EGFR^high^ GSCs are characterized by enhanced tumorigenic potential and highly invasive behavior, while EGFR^low^ GSCs form tumors with low efficiency and need to upregulate EGFR to be tumorigenic^26^. Gene expression profiling revealed that pro-invasive and angiogenic genes were overexpressed in xenografts derived from cells with high or low EGFR expression, respectively^26^. Interestingly, in EGFR^low^ cells, we found activation of the apelin signaling pathway, which is implicated in angiogenesis^50,51^. GSCs with different EGFR statuses responded differently to pharmacologic inhibition of EGFR^26^.

AG1478 (a specific EGFR tyrosine kinase inhibitor) efficiently reduced EGFR activation in EGFR^high^ GSCs, but downstream PI3K-AKT signaling pathways were not affected. Treatment with AG1478 reduced the growth of EGFR^high^ cells, although there was no activation of Caspases (Fig. S4e–g), which indicates that AG1478 reduced cell proliferation and induced cell cycle arrest in the G1 phase, as reported elsewhere^52^. In fact, several agents (antibodies as well as small molecules) targeting EGFR in glioblastomas have already been tested in patients, but targeting EGFR has not provided therapeutic benefits^53,54^. This could explain the lack of impact of AG1478 treatment on TMZ-induced apoptosis in EGFR^high^ BMP4-differentiated cells, for which the downregulation of AKT is crucial. The EGFR-independent pathway may maintain stimulation of the PI3K-AKT signaling pathway. We and others previously reported an impact of integrin or JAK signaling in glioma intracellular signaling^55,56^, and other receptor tyrosine kinases^57–60^ may be involved. Clark et al.^57^ demonstrated a compensatory activation of EGFR-related family members (ERBB2 and ERBB3) that enabled glioma stem cell proliferation, suggesting that simultaneous blockade of multiple ERBB family members may be required for more efficacious GBM therapy. Other studies demonstrated that repression of AKT signaling and induction of apoptosis required concurrent inhibition of both EGFR and InsR (insulin receptor)/IGF-1R (insulin-like growth factor-1 receptor) signaling. Combining gefitinib (a specific EGFR inhibitor) and OSI-906 (a dual inhibitor of InsR/IGF-1R) was more effective than either agent alone in the inhibition of subcutaneous glioblastoma xenografts^58^. Tumors can evade EGFR inhibition through the upregulation and phosphorylation of MET tyrosine kinase receptor^59^ and by activation of the FGFR (fibroblast growth factor receptor)-ERK (extracellular signal-regulated kinases)-SPRY2 (Sprouty 2) signaling axis^60^. This dynamic rewiring of signaling processes that is observed in glioma cells could explain our failure in the inhibition of key downstream EGFR signaling components, such as AKT and FOXO3a, after using AG1478.

Several obstacles and failures of BMP-induced differentiation therapy for GBM have been recently reported^51^. Our findings help to comprehend why BMP-induced differentiation of GSCs does not improve therapeutic efficacy and why such therapy should be used with caution, especially for GBMs with low expression of EGFR. In summary, our results demonstrate that enhanced EGFR signaling causes re-entry of BMP4-differentiated EGFR^high^ GSCs into the cell cycle, and only in such cells does TMZ trigger BIM-dependent apoptosis. This result is accomplished through reduction of PI3K/AKT signaling and activation of the FOXO3a/BIM axis, which is inactivated in EGFR^low^ cells. Our findings support the idea of evaluating the EGFR status in GSCs when choosing therapeutic strategies aimed at eliminating cancer stem cells.

## Supplementary information

supplementary information
