# Supplementary material for: EGFR/FOXO3a/BIM signaling pathway determines chemosensitivity of BMP4-differentiated glioma stem cells to temozolomide
Source: Exp Mol Med. 2020 Aug 12;52(8):1326–40. doi: 10.1038/s12276-020-0479-9 (PMC8080762; doi:10.1038/s12276-020-0479-9)
Supplement: Supplementary file 1 — supplementary information [file 12276_2020_479_MOESM1_ESM.pdf]

EGFR/FOXO3a/BIM signalling pathway determines chemosensitivity of BMP4-differentiated glioma stem cells to temozolomide

Iwona Anna Ciechomska[1], Bartlomiej Gielniewski[1], Bartosz Wojtas[1], Bozena Kaminska[1#], Jakub Mieczkowski[1#]

[1]Laboratory of Molecular Neurobiology, Neurobiology Center, Nencki Institute of Experimental Biology PAS, 3 Pasteur St, 02-093 Warsaw, Poland

Supplementary Information

Figure S1

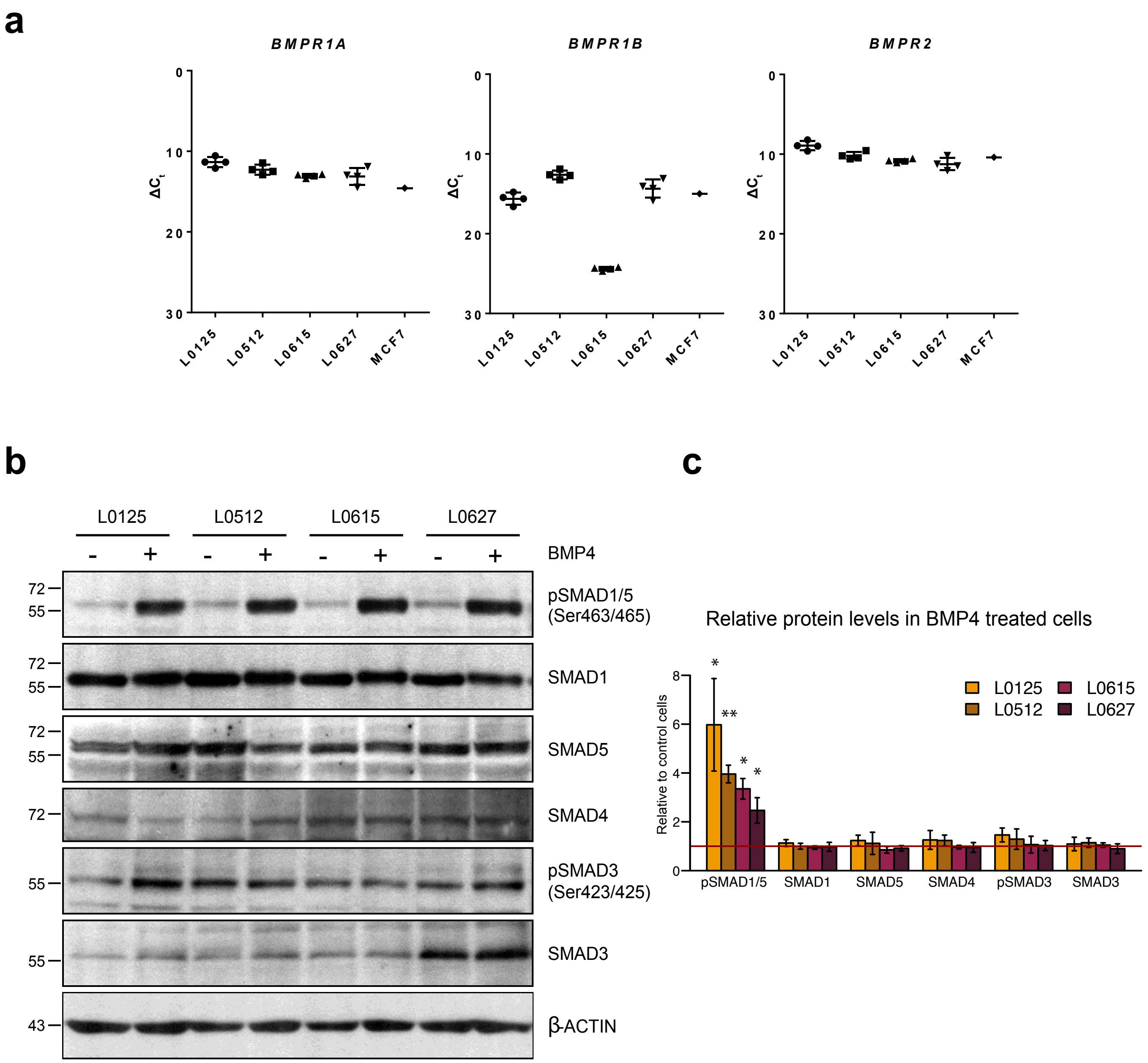

Figure S1. Activation of BMP signaling pathway.
(a) Quantitative analysis of *BMPR1A*, *BMPR1B*, *BMPR2* gene expression in human glioma spheres as determined by qPCR. MCF7 cell line was used as a positive control. **(b)** Western blot analysis of SMAD signalling cascade in GSCs. Immunoblot shows the levels of phospho-SMAD1/5 (Ser463/465), phospho-SMAD3 (Ser423/425), SMAD1, SMAD3, SMAD4 and SMAD5 in glioma spheres treated with 100 ng/ml BMP4 for 90 min. Antibody against β-Actin was used to ensure an equal protein loading. **(c)** Densitometric analysis of the blots and quantification of the results from three independent experiments is shown; bars represent means ± SD of phospho-SMAD1/5 (Ser463/465), phospho-SMAD3 (Ser423/425), SMAD1, SMAD3, SMAD4, SMAD5 levels normalized to β-Actin and then to the control (*$P < 0.05$, **$P < 0.01$, t-test).

Figure S2

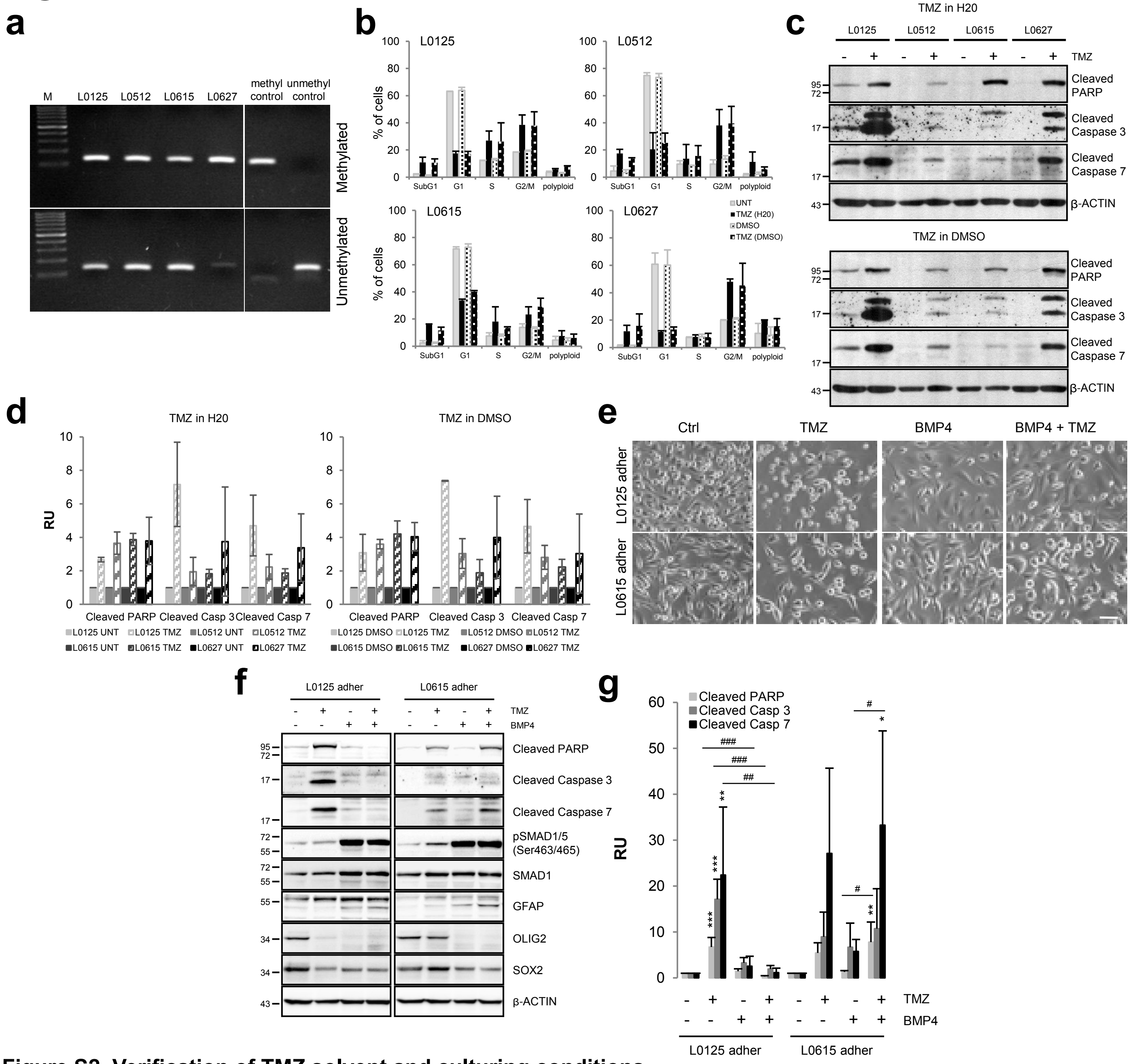

Figure S2. Verification of TMZ solvent and culturing conditions.

(a) Methylation status of *MGMT* gene promoter in GSC cell lines was determined by methylation-specific PCR assay. The PCR products were separated on 1.5 % agarose gel, visualized by SimplySafe staining. The methylated, as well as unmethylated, bisulfite converted DNA were used as a positive and negative controls for methylated or unmethylated templates, respectively. A 100-bp marker ladder was loaded to estimate molecular size. Representative results of three independent experiments ae shown. **(b)** The effect of TMZ on the cell cycle. Cell cycle phases distribution in GSCs after 72 h TMZ (500 µM) treatment was carried out using FACS calibur flow cytometer by DNA staining with propidium iodide. Results are presented as means ± SD of two independent experiments. **(c)** Western blot analysis of biochemical markers of apoptosis. Immunoblot shows the levels of Cleaved Caspase 3, Caspase 7 and PARP in control and TMZ-treated glioma cells. Antibody against β-Actin was used to ensure an equal protein loading. **(d)** Densitometric analysis of the blots and quantification of the results from two independent experiments is shown; bars represent means ± SD of the cleaved caspases, and cleaved PARP levels normalized to the control and then to β-Actin. **(e)** Representative microphotographs of untreated adherent GSCs or treated with drugs. Picture were taken after 4 days of BMP4 (100 ng/ml) treatment and additional 3 days with or without TMZ (500 µM). Scale bars represent 50 µm. **(f,g)** Western blot (f) and the densitometric analysis (g) of biochemical markers of apoptosis. Immunoblots show the levels of Cleaved Caspase 3, Caspase 7 and PARP, as well as phospho-SMAD1/5 (Ser463/465), SMAD1, GFAP, OLIG2, SOX2 in untreated, BMP4- and/or TMZ-treated adherent GSCs. (f) Cell lines and combination of treatments are indicated on the top. Antibody against β-ACTIN was used to ensure an equal protein loading. (g) Each bar represent mean ± SD of three independent experiments. *P < 0.05, **P < 0.01,***P < 0.001 compared to untreated control; #P < 0.05, ##P < 0.01,###P < 0.001 BMP4 or TMZ-treated cells versus cells treated with both drugs (post-hoc test in ANOVA).

Figure S3

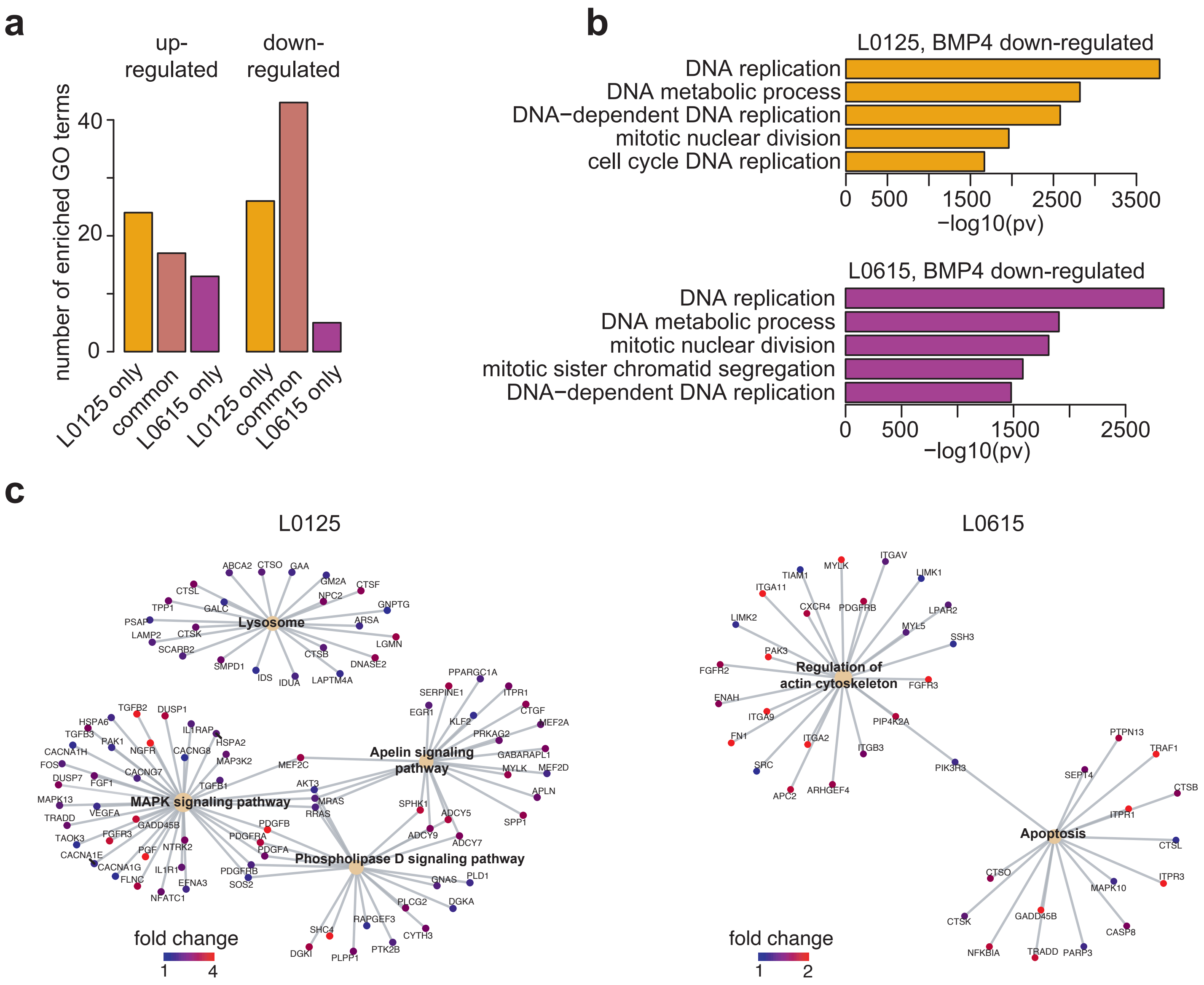

Figure S3. Gene ontology analysis and genes belonging to specifically up-regulated signalling pathways
(a) Number of Gene Ontology terms significantly enriched in the sets of differentially down- or up-regulated genes. Left bars correspond to sets of up-regulated genes, while right bars correspond to sets of down-regulated genes. Yellow and purple bars depict results specific for to L0125 and L0615 cell lines, respectively. Light purple bars (second and fifth from the left) show number of non-specifically enriched pathways. **(b)** Top 5 Gene Ontology terms enriched in sets of genes significantly down-regulated genes after BMP4-treatment. Yellow panel corresponds to L0125 cell line and purple panel corresponds to L0615 cell line. X-axes on both plots represent Benjamini and Hochberg adjusted P-values computed with Fisher's exact test. **(c)** Networks visualizing signalling pathways specifically up-regulated in L0125 (left) and L0615 (right) cell lines. These results correspond to Figure 3f.

Figure S4

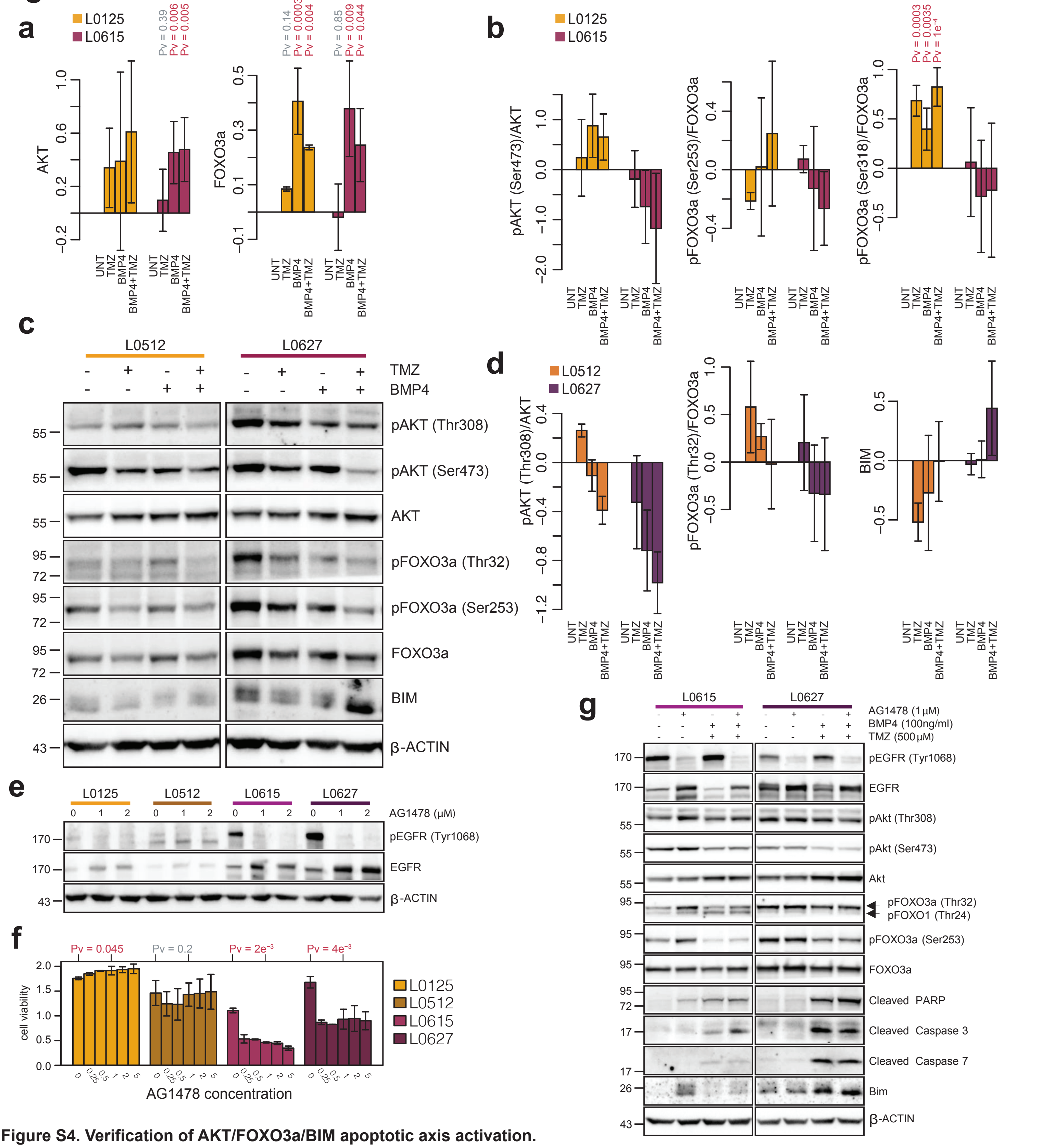

Figure S4. Verification of AKT/FOXO3a/BIM apoptotic axis activation.
(a) A densitometric analysis of total AKT and FOXO3a levels. These results correspond to Figure 4c. Each bar and whisker represent the mean ± SD of three independent experiments. The total proteins levels were normalized to β-ACTIN. See legend for Figure 1f for more details on statistical analysis. **(b)** The densitometric analysis of phospho-AKT (Thr473) and phospho-FOXO3a (Ser253 and Ser318). These results correspond to Figure 4c. Each bar and whisker represent the mean ± SD of three independent experiments (log-scale). The levels of phosphorylated proteins were normalized to corresponding total protein levels. See legend for Figure 1f for more details. **(c)** Western blot analysis of total- and phospho-AKT, total- and phospho-FOXO3a and BIM levels upon exposure to TMZ, BMP4 or both drugs. β–ACTIN was used as a loading control. **(d)** The densitometric analysis of phospho-AKT (Thr308) phospho-FOXO3a (Thr32) and BIM levels performed with L0512 (EGFR^{low}, orange bars) and L0627 (EGFR^{high}, purple bars). Each bar and whisker represent the mean ± SD of two independent experiments (log-scale). The levels of phosphorylated proteins were normalized to corresponding total protein levels and the BIM levels were normalized to β-ACTIN. See legend for Figure 1f for more details. **(e)** Western blot analysis of phospho-EGFR (Tyr1068) and EGFR in untreated and glioma spheres treated with 1, 2 mM AG1478 for 3 days. β-ACTIN was used as a loading control. **(f)** The effects of different doses of AG1478 on viability of EGFRlow and EGFRhigh cells. The cells were treated for 3 days. The significance of difference was tested with paired t-test (n=3) **(g)** Western blot analysis of EGFR signaling pathway and biochemical markers of apoptosis. Immunoblots show the levels of total- and phospho-EGFR, total- and phospho-AKT, total- and phospho-FOXO3a, Cleaved Caspase 3, Caspase 7 and PARP, as well as BIM in untreated and treated EGFRhigh cells. Cells were exposure to AG1478 alone for 3 days, or first BMP4-differentated (for 4 days) then treated with TMZ alone (for 3 days) or with both drugs AG1478 and TMZ (for 3 days). Detection of β-ACTIN ensured an equal protein loading.

Supplementary Table 1

Sequences of primers used in this work.

BMPR1A_qPCR	CAGGTTCCTGGACTCAGCTC	CTTTCCTTGGGTGCCATAAA-
BMPR1B_qPCR	AAAGGTCGCTATGGGGAAGT	GCAGCAATGAAACCCAAAAT
BMPR2_qPCR	GCTAAAATTTGGCAGCAAGC	CTTGGGCCCTATGTGTCACT
SOX2_qPCR	GGGGAAAGTAGTTTGCTGCC	CGCCGCCGATGATTGTTATT
PROM1_qPCR	TGGATGCAGAACTTGACAACGT	ATACCTGCTACGACAGTCGTGGT
OLIG2_qPCR	TCGCATCCAGATTTTCGGGT	AAAAGGTCATCGGGCTCTGG
NESTIN_qPCR	CAAGACTTCCCTCAGCTTTCAG	AGGTGTCTCAAGGGTAGCAG
GFAP_qPCR	TCCTGGAACAGCAAAACAAG	CAGCCTCAGGTTGGTTTCAT
TUBB3_qPCR	GTACGTGCCTCGAGCCATTCT	CGTGTAGTGACCCTTGGCCC
18S_qPCR	CGGACATCTAAGGGCATCAC	AACGAACGAGACTCTGGCAT
Methylated MGMT_MS-PCR	TTTCGACGTTCGTAGGTTTTCGC	GCACTCTTCCGAAAACGAAACG
Unmethylated MGMT_MS-PCR	TTTGTGTTTTGATGTTTGTAGGTT TTTGT	AACTCCACACTCTTCCAAAAACA AAACA
